# Novel Bio-Based Epoxy Thermosets Based on Triglycidyl Phloroglucinol Prepared by Thiol-Epoxy Reaction

**DOI:** 10.3390/polym12020337

**Published:** 2020-02-05

**Authors:** Dailyn Guzmán, David Santiago, Àngels Serra, Francesc Ferrando

**Affiliations:** 1 Eurecat–Chemical Technology Unit, c/Marcel·lí Domingo 2, Edif. N5, 43007 Tarragona, Spain; david.santiago@eurecat.org; 2 Department of Analytical and Organic Chemistry, Universitat Rovira i Virgili, c/Marcel·lí Domingo 1, Edif. N4, 43007 Tarragona, Spain; angels.serra@urv.cat; 3 Department of Mechanical Engineering, Universitat Rovira i Virgili, Av. Països Catalans 26, 43007 Tarragona, Spain; f.ferrando@urv.cat

**Keywords:** epoxy thermoset, phloroglucinol, thiol-epoxy, latent catalyst, sustainability

## Abstract

The pure trifunctional glycidyl monomer from phloroglucinol (3EPO-Ph) was synthesized and used as feedstock in the preparation of novel bio-based thermosets by thiol-epoxy curing. The monomer was crosslinked with different commercially available thiols: tetrafunctional thiol (PETMP), trifunctional thiol (TTMP) and an aromatic dithiol (TBBT) as curing agents in the presence of a base. As catalyst, two different commercial catalysts: LC-80 and 4-(*N*,*N*-dimethylamino) pyridine (DMAP) and a synthetic catalyst, imidazolium tetraphenylborate (base generator, BG) were employed. The curing of the reactive mixtures was studied by using DSC and the obtained materials by means of differential scanning calorimetry (DSC), thermogravimetric analysis (TGA) and dynamic mechanical thermal analysis (DMTA). The results revealed that only the formulations catalyzed by BG showed a latent character. Already prepared thermosetting materials showed excellent thermal, thermomechanical and mechanical properties, with a high transparency. In addition to that, when compared with the diglycidyl ether of bisphenol A (DGEBA)/PETMP material, the thermosets prepared from the triglycidyl derivative of phloroglucinol have better final characteristics and therefore this derivative can be considered as a partial or total renewable substitute of DGEBA in technological applications.

## 1. Introduction

Nowadays, polymeric eco-friendly materials have been extensively studied as substitutes of commercial materials derived from oil to be used in future industrial applications. They involve the use of starting compounds based on renewable resources of great natural abundance, low cost and safe for human health [1,2].

There are many epoxy derivatives synthesized from natural resources that have been studied for the preparation of epoxy thermosets [3,4,5]. They have been used as partial or total substitutes of oil-derived epoxy resins such as diglycidyl ether of bisphenol A (DGEBA), which is classified as unsafe because it can act as an endocrine disruptor and therefore is not suitable for applications related to the food industry. There is a great variety of bio-based monomers and among them we can mention vanillin [6,7], itaconic acid [8], cardanol [9], furan derivatives [10], sugars [11] or fatty acids [12,13]. However, the best option in the preparation of renewable thermosetting materials that can substitute DGEBA thermosets are those having phenolic groups because the rigidity of the aromatic structure allows reaching excellent thermal and mechanical performances [14,15].

In a previous study of our group, two new epoxy monomers based on eugenol were synthetized and used to prepare epoxy thermosetting materials using thiols as curing agents in both cases. In this way it was possible to obtain thermosets with similar properties or even better than DGEBA materials depending on the functionality of the starting monomers used. The results put in evidence that these phenolic compounds are a good renewable alternative to commercial oil-derived resins [16,17].

Phloroglucinol, with three phenolic groups in its compact structure, is one of the major components present in *Ecklonia cava* (brown algae). It can also be obtained by a synthetic route from benzene [18,19]. The most important application of this compound is in the pharmaceutical industry because it has excellent antispasmodic properties [20,21]. Due to its great biocompatibility and good chemical characteristics, this aromatic compound could also be used as feedstock for the preparation of epoxy thermosets with excellent final properties [22].

Several authors have reported the preparation of thermosets starting from triglycidyl phloroglucinol resin (3EPO-Ph). Recently, Noè et al. [23] prepared epoxy thermosets by cationic photopolymerization of 3EPO-Ph resin using a 4% wt. of photoinitiator. The new material presented an unexpected low *T*_g_ value (48 °C), probably due to the presence of oligomers in the resin which reduced the crosslinking density that can be achieved in cationic homopolymerization processes. In another study, the 3EPO-Ph resin was derivatized to prepare a new diepoxy compound with one phosphate group. The presence of phosphorous in the resin structure allowed increasing the flame retardancy and enhanced other final characteristics of the thermosets prepared [24]. In a similar work, all the epoxy groups of 3EPO-Ph resin were transformed into phosphate groups and this new compound was used as additive for commercial epoxy resins. The final materials showed excellent thermal and mechanical characteristics and the addition of the triphosphate improved considerably the flame retardancy of the epoxy thermosets [25].

The epoxy resin derived from phloroglucinol cured with dicyandiamide has also been used in the preparation of materials able to be applied in the food industry [26]. The materials exhibited excellent thermal stability and a high *T*_g_ (187 °C). In addition, they showed high glass adhesion and good mechanical performance. These results demonstrate that this type of materials are promising alternatives for DGEBA-based thermosets.

**Figure 1:**
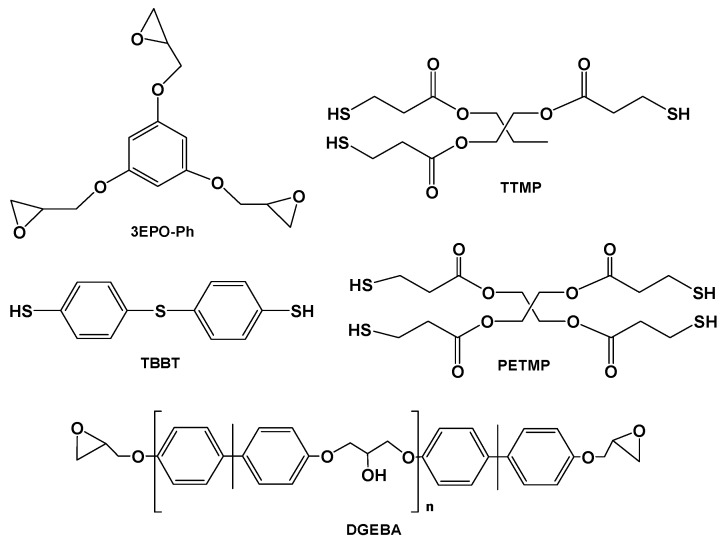
Chemical structures of the monomers used.

The present study proposes the preparation of a pure triglycidyl phloroglucinol monomer which has a high functionality and a compact structure (3EPO-Ph) that will be later cured by a thiol-epoxy click reaction in the presence of a base. As curing agent, several thiols were selected. We chose commercially available thiols of different functionality, from 2 to 4. The structures of the phloroglucinol derivative synthesized and of the three thiols selected are presented in Figure 1.

The use of click reactions for the preparation of thermosetting materials involves an eco-friendly technology due to the lower amount of waste originated, the low energy consumption, the completeness of the reaction process and the high yield without the formation of by-products that leads to the formation of highly homogenous three-dimensional network structures. Finally, one of the most important characteristics is the possibility to perform these reactions in air atmosphere, which is of the utmost importance in technological applications.

Finally, it is worth mentioning that the latency of the formulation, which allows its storage during a certain period before application and simplify the technology associated to the industrial fabrication of pieces and complex devices, has also been considered in the present study. The use of several amines as basic catalyst, such as *N*-benzyldimethylamine, in thiol-epoxy formulations, leads to an excessive fast reaction that makes difficult to industrially apply these formulations. For this reason, the use of several tertiary amines which showed a certain latent character (LC80 and DMAP), has been essayed together with a thermally activated base generator catalyst. This base generator synthesized by us can be not only photochemically but also thermally activated as it was previously demonstrated [27].

## 2. Experimental

### 2.1. Materials

Phloroglucinol, benzyl triethylammonium chloride (TEBAC), epichlorohydrin (EPC), 4,4′-thio-bisbenzenethiol (TBBT), pentaerythritol tetrakis(3-mercaptopropionate) (PETMP), trimethylol-propane tris(3-mercaptopropionate) (TTMP), 4-(*N*,*N*-dimethylamino)pyridine (DMAP), 1-methyl-imidazole (1-MI), benzyltriethylammonium chloride (TEBAC) and sodium tetraphenylborate were purchased from Sigma-Aldrich (Saint Louis, MO, USA) and were used without further purification. Technicure^®^ LC-80 (encapsulated imidazole) was obtained from AC Catalyst (Linden, NJ, USA). Diglycidylether of bisphenol A (DGEBA, Araldite GY240 (Huntsman Advanced Materials, Everberg, Belgium), epoxy equivalent 182 g/eq) was dried at 80 °C under vacuum for 6 h. Inorganic salts and bases were purchased from Scharlab (Barcelona, Spain). Chloroform, hexane, methylene chloride and ethyl acetate from Carlo Erba (Barcelona, Spain) have been used as received.

### 2.2. Preparation of Starting Products 

#### 2.2.1. Synthesis of Triepoxy Phloroglucinol (3EPO-Ph)

Phloroglucinol (10.49 g, 0.06 mol), epichlorohydrin (116.85 g, 1.26 mol) and benzyltriethyl-ammonium chloride (TEBAC, 2.60 g, 0.01 mmol) were stirred in a 500 mL flask at 100 °C for 4 h. The mixture was cooled down to 30 °C and then an aqueous solution of 20% NaOH (90 mL) and TEBAC (2.60 g) were added and maintained under stirring for 90 min, after that, ethyl acetate (60 mL) was added to the mixture for dilution. The phases were separated, and the organic layer was washed twice with water and dried with magnesium sulphate. The solvent and excess epichlorohydrin were eliminated in a rotary evaporator at 60 °C. The resin obtained was purified by silica-gel chromatography using ethyl acetate/hexane 6/4. The monomer obtained is a white powder with a 60% (10.58 g) yield. Melting temperature: 53 °C (determined by DSC). ^1^H-NMR (CDCl_3_, δ in ppm): 6.1 s (Ar, 3H), 4.2 dd (–CH_2_–O–, 3H), 3.99 dd ((–CH_2_–O–, 3H), 3.3 m (CH epoxy ring, 3H), 2.9 m (CH_2_ epoxy ring, 3H) and 2.7 m (CH_2_ epoxy ring, 3H). ^13^C-NMR (CDCl_3_, δ in ppm): 160.2, 94.8, 68.8, 50.0 and 44.5. FT-IR (ATR): 3064, 3006, 2924, 2872, 2837, 1592, 1446, 1427, 1342, 1248, 1166, 1151, 1129, 1059, 984, 904, 858, 831 cm^−1^.

#### 2.2.2. Synthesis of the Base Generator (BG)

The latent catalyst BG was synthesized following a procedure described in the literature [28]. 1-MI (0.82 g, 10 mmol) was dissolved in an acidic solution (1 mL conc. HCl/2.6 mL H_2_O) under magnetic stirring at room temperature and then, a solution of NaBPh_4_ (3.74 g, 11 mmol) in H_2_O was added slowly. The precipitate was filtered and washed several times with water and MeOH. The solid obtained was recrystallized from a 4:1 mixture of MeOH/CHCl_3_. The product was dried at 40 °C in a vacuum oven. The decomposition temperature determined by DSC was 90 °C.

### 2.3. Preparation of the Curing Mixtures

The mixtures were prepared by mixing stoichiometric amounts of epoxide/SH groups (1:1) of 3EPO-Ph with the aliphatic tetrafunctional (PETMP) and trifunctional (TTMP) thiols. The insolubility of the aromatic thiol (TBBT) in the formulation required the addition of TTMP to reach a homogeneous mixture. Thus, this mixture was prepared with 40 % of TBBT and 60 % TTMP in mol, with the stoichiometric amount of 3EPO-Ph. To all the formulations 1 phr (parts of catalyst per hundred parts of thiol compound) of the three different catalysts tested was added. The catalyst selected was LC-80 (encapsulated imidazole), 4-(*N*,*N*-dimethylamino)pyridine (DMAP), and 1-methylimidazolium tetrafluoroborate (BG). All the 3EPO-Ph/thiol formulations were prepared in solution by adding a few drops of dichloromethane and homogenizing with a spatula. The solvent was evaporated in vacuum. A formulation with triepoxy derivative from phloroglucinol without purification by column chromatography (3EPO-Ph resin) and PETMP was prepared to study the influence of impurities in the curing process and final properties of the thermoset obtained. Another formulation, DGEBA/PETMP, was prepared for a comparative study of this commercial resin with the renewable triepoxy derivative.

### 2.4. Characterization Techniques

^1^H-NMR and ^13^C-NMR spectra were registered on a Gemini 400 spectrometer (Varian, Lake Forest, CA, USA). CDCl_3_ was used as the solvent. For internal calibration the solvent signal corresponding to CDCl_3_ was used: δ (^1^H) = 7.26 ppm, δ (^13^C) = 77.16 ppm.

The study of the epoxy-thiol thermal curing was performed by differential scanning calorimetry (DSC) in a DSC+3 Stare system apparatus (Mettler, Columbus, OH, USA). The calorimeter was calibrated using an indium standard (heat flow calibration) and an indium-lead-zinc standard (temperature calibration). For dynamic studies, a flow of N_2_ at 50 mL/min was used and the weight of the samples for the analysis was 10 mg. These studies were performed in the temperature range of 30–275 °C, with a heating rate of 10 K/min.

The glass transition temperatures (*T*_g_s) of the samples after curing were determined in dynamic scans at 20 °C/min from room temperature to 150 °C. The *T_g_*s of the final thermosets were determined after two consecutive heating dynamic scans at 20 °C/min starting at −20 °C to delete the thermal history. The *T*_g_ value was taken as the middle point in the heat capacity step of the glass transition.

A FT/IR-6700 spectrometer (Jasco, Hachioji, Tokyo, Japan) equipped with an attenuated-total-reflectance accessory with a diamond crystal (Golden Gate heated single-reflection diamond ATR, Specac-Teknokroma, Barcelona, Spain) was used. The FTIR spectra were recorded in the wavenumber range between 4000 and 600 cm^−1^ with a resolution of 4 cm^−1^ and averaged over 20 scans.

The thermal stability of the thermosets was studied by thermogravimetric analysis (TGA) using a Mettler TGA/SDTA 851e thermobalance (Columbus, OH, USA). All experiments were conducted under inert atmosphere (N_2_ at 100 mL/min). Pieces of the cured samples with an approximate mass of 8 mg were degraded between 30 and 600 °C at a heating rate of 10 K/min.

Dynamic mechanical thermal analyses (DMTA) were carried out with a DMA Q800 analyzer (TA Instruments, New Castle, DE, USA). Thiol-epoxy formulations were isothermally cured in a prismatic mold in an optimized schedule as follows: (a) 135 °C for 2 h, with a post curing at 175 °C for 3 h in case of 3EPO-Ph/TTMP, (b) 120 °C for 2 h with a post curing at 160 °C for 3 h for 3EPO-Ph/PETMP, (c) 100 °C for 1 h and 135 °C for 1 h with a post curing at 175 °C for 3 h for 3EPO-Ph/TTMP-TBBT, (d) 110 °C for 2 h with a post curing at 165 °C for 3 h for 3EPO-Ph resin/PETMP and (e) 130 °C for 2 h with a post curing at 190 °C for 3 h for DGEBA/PETMP. Three-point bending clamp was used on the prismatic rectangular samples. The samples were analyzed at 3 K/min from 30 to 100 °C at a frequency of 1 Hz with a deformation of 0.1%.

Young’s modulus was determined under flexural conditions at 30 °C with the same clamp and geometry samples applying a force ramp at constant load rate of 1 N/min from 0.005 N to 18 N. Three samples of each material were analyzed, and the results were averaged. Stress-strain tests were performed with the film-tension clamp in force-controlled mode. Dog-bone samples were used at a force rate of 1 N/min and the mean values of at least three different samples were reported. 

Microindentation hardness was measured with a 401 MAV device (Wilson Wolpert, Aachen, Germany) following the ASTM E384-16 standard procedure. For each material 10 determinations were made with a confidence level of 95%. The Vickers microindentation hardness number (*HV*) was calculated by the following equation:

(1) $$HV=\frac{1.8544\;F}{d^{2}}$$

where, *F* is the load applied to the indenter in kgf and *d* is the arithmetic mean of the length of the two diagonals of the surface area of the indentation measured after load removal in mm.

## 3. Results and Discussion

### 3.1. Synthesis and Characterization of the Starting Compounds

**Scheme 1:**
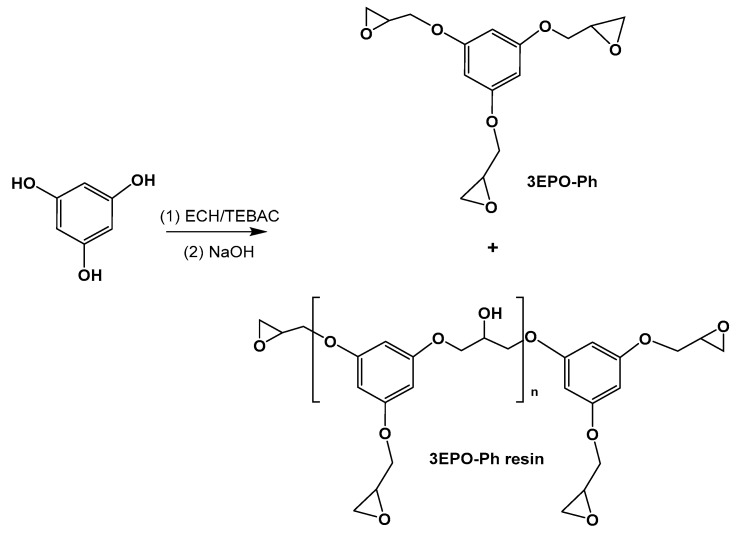
Synthetic pathway for the preparation of the triglycidylether derivative of phloroglucinol.

The triglycidyl derivative from phloroglucinol was synthesized by reacting the phenolic compound with a large excess of epichlorohydrin in the presence of a transfer catalyst, as it was previously reported [22]. After that, the treatment in alkaline medium helps to cyclize the formed chlorohydrine intermediates (Scheme 1). This synthetic procedure can lead to a partial oligomerization, as occurs in the preparation of DGEBA resins.

**Figure 2:**
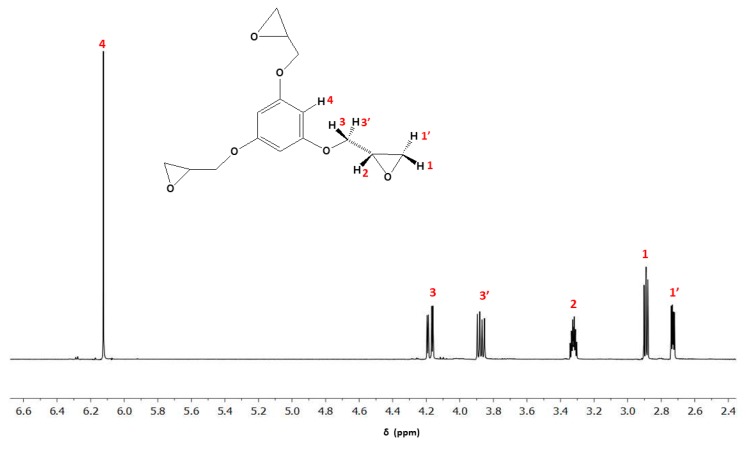
^1^H-NMR spectrum of 3EPO-Ph monomer in CDCl_3_.

**Figure 3:**
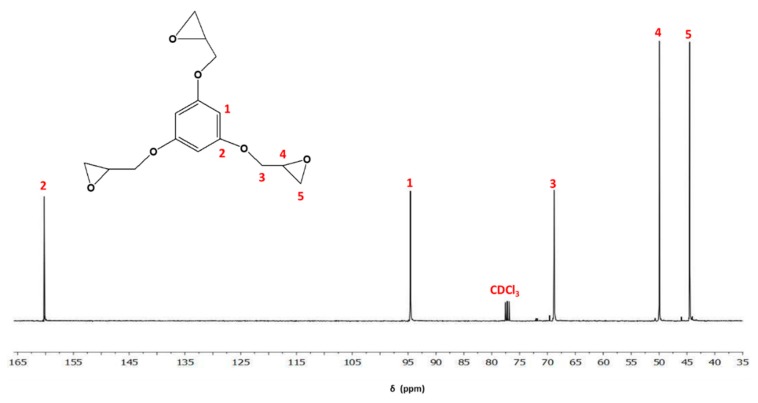
^13^C-NMR spectrum of 3EPO-Ph monomer in CDCl_3_.

Some authors employed the 3EPO-Ph resin as starting material for the preparation of thermosets. However, the presence of oligomers that increases the epoxy equivalent affects the crosslinking density and rigidity, reducing the *T*_g_s and mechanical properties of the final materials [23]. For this reason, in this work, we have started from pure 3EPO-Ph monomer obtained by purification of the crude synthetic product by chromatographic column. The high purity of the compound was confirmed by NMR (see Figure 2 and Figure 3) and FT-IR spectroscopy. The product was a white solid with a melting point of 53 °C determined by DSC, while the product before the purification was a yellowish viscous liquid.

In Figure 2 we can clearly observe in the ^1^H-NMR spectrum the presence of aromatic protons and the protons of the glycidyl ether groups directly attached to the aromatic ring. The highly symmetric character of the structure leads to a quite simple spectrum. From 4.3 to 2.6 ppm, it can be observed the signals corresponding to the glycidyl groups with five unequivalent protons due to the presence of the methine asymmetric carbon that leads to the methylene protons to be diastereotopic among them. The protons have been assigned according to their chemical shifts and the coupling constants, related to the torsional angle. The assignments have been done in the chemical structure included in the figure. The integration of the signals, the splitting and the chemical shifts support the structure for 3EPO-Ph.

Figure 3 shows the ^13^C-NMR spectrum of the epoxy derivative with the corresponding assignments, which were mainly done by comparison with similar structures. It shows only five signals because of the molecule’s high symmetry. The signals at 160.2 and 94.8 ppm correspond to the aromatic ring carbons, while the signals from 68.8 to 44.5 ppm are assigned to glycidyl carbons.

The absence of significant impurities in both NMR spectra, compared to what was reported in the literature [23] should be noted since it puts in evidence the monomeric characteristics and the high purity of the synthesized product. The characterization of 3EPO-Ph by FTIR spectroscopy showed the characteristic absorptions corresponding to its chemical structure. In addition to the typical aromatic and ether bands, the most relevant band appears at 904 cm^−1^ that confirms the presence of the glycidyl groups.

### 3.2. Study of the Curing Process

**Figure 4:**
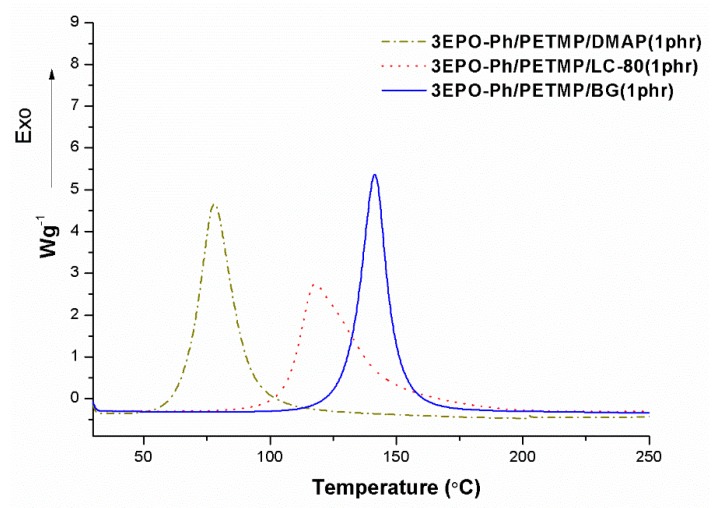
Calorimetric curves of the initial mixture 3EPO-Ph/PETMP formulations with 1 phr of the different catalysts.

We selected thiols as the curing agents to get thermosets from 3EPO-Ph. The curing with thiols requires the presence of a base or nucleophile to increase the reactivity of thiols [16,17]. In the present case, we selected DMAP, LC-80 (which is an encapsulated imidazole) and BG (which is an imidazolium tetraphenylborate salt). It has been described that LC-80 and BG have latent characteristics, which is highly important in the preparation of one-pot formulations and to facilitate the application of these formulations. To check the effect of these catalysts in the curing of 3EPO-Ph/thiol stoichiometric formulations we firstly studied the curing evolution by calorimetry in dynamic conditions. Figure 4 shows the DSC thermograms of the dynamic curing at 10 °C/min for 3EPO-Ph/PETMP mixtures with 1 phr of DMAP, LC-80 and the previously synthesized latent catalyst (BG).

**Scheme 2:**
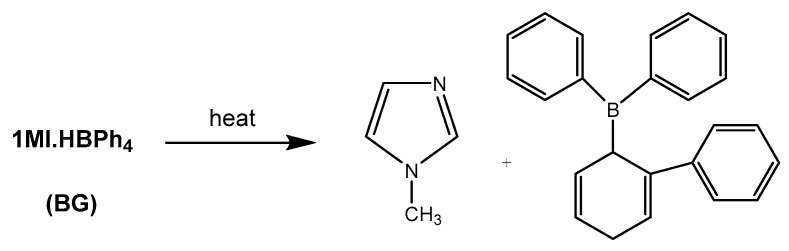
1MI.HBPh_4_ (BG) and the compounds formed in its thermal decomposition.

As it can be observed, in case of the mixtures containing DMAP and LC-80 the curing begins at lower temperatures and the exotherms are broader and flatter that the one containing BG, which indicates less stability of the one-pot formulation and slower curing rate once the curing begins. The decomposition of BG at a defined temperature releases 1-MI, which is the active specie responsible of the thiolate formation (see Scheme 2). The exotherm is in this case sharp, indicating the higher latency of BG in reference to the other basic catalysts and therefore it is the best option for the preparation of thermosets from 3EPO-Ph formulations. It should be commented that LC-80 led to high latency in DGEBA/thiol formulations [29] producing the curing at high temperatures with a high rate after initiated. This behaviour has not been observed in the present study and can be explained by the loss of stability of the capsule in the reactive medium.

Table 1 shows the values of enthalpy released and the glass transition temperatures obtained by DSC in the curing of the mixtures with the different catalysts.

The values of the enthalpy are similar for all formulations with values around 130 KJ/eq indicating that the degree of curing achieved is also similar. The glass transition temperatures of the cured materials show only small differences among them and they are well above room temperature.

**Figure 5:**
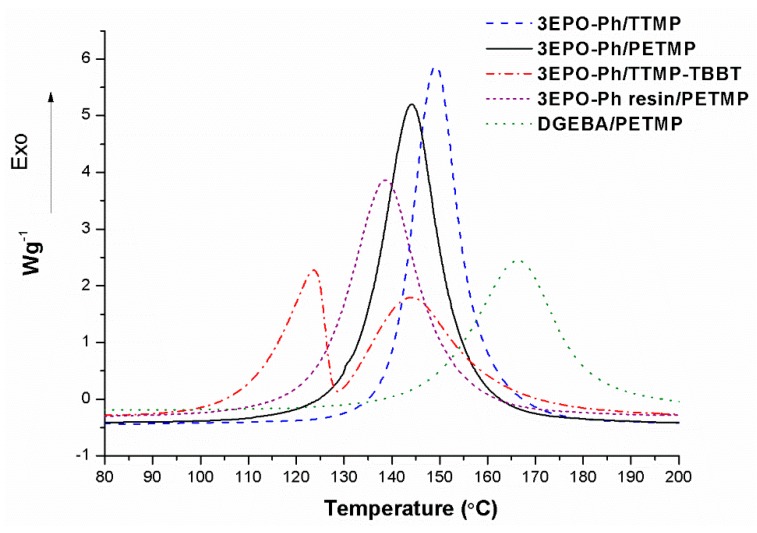
Curing exotherms of the stoichiometric 3EPO-Ph/thiol formulations with. 1 phr of the BG as the catalyst and curves of the 3EPO-Ph resin and DGEBA, both with PETMP.

Once BG was selected as the catalyst, the evolution of the curing process for different stoichiometric mixtures of 3EPO-Ph with different thiols and 1 phr of BG was investigated by DSC. The resulting curves are represented in Figure 5 and the data extracted are collected in Table 2. To make a comparison, the calorimetric curve and the corresponding DGEBA/PETMP formulations’ data have also been added.

The thermograms in Figure 5 show significant differences in reactivity among all the thiol-epoxy formulations. As we can see, all formulations have a unimodal curve with the exception of the formulation including a mixture of aliphatic and aromatic thiols (TTMP/TBBT) that shows a clear bimodal shape. In this curing process, there is a curve at high temperature attributable to the 3EPO-Ph/TTMP reaction and another one at lower temperature due to the reaction of epoxide with the aromatic dithiol (TBBT) which has a higher nucleophilic character. The higher reactivity of the aromatic thiols in comparison to the aliphatic ones leads to a curing at lower temperatures in a broad temperature range without any sign of latency. In Figure 5, we can also see that reactivity of the formulations containing PETMP are affected by the presence of impurities in the epoxy compound. The curing exotherm corresponding to the 3EPO-Ph resin begins at a lower temperature resulting in a broader and lower curve than in the case of the formulation prepared from pure 3EPO-Ph. The curing of the formulation with DGEBA takes place at a higher temperature indicating a lower reactivity of this compound in comparison to the phloroglucinol derivative.

The degree of curing achieved for the formulations is related to the enthalpy released by epoxy equivalent. As we can see in Table 2, mixtures 3EPO-Ph/TTMP and 3EPO-Ph/PETMP show some variations in the enthalpy released per gram but the values for epoxy equivalent are similar, indicating that the same degree of curing was achieved. Considering that both formulations’ enthalpy values range between 125–130 kJ/eq we can state that the degree of curing was complete and this fact highly agrees with which was described previously [30]. However, the formulations containing the aromatic thiol or the 3EPO-Ph resin show lower enthalpy values than expected. The presence of aromatic moieties in the thiol structure could diminish the enthalpy released by introducing some topological restrictions to complete the curing. In the case of the formulation 3EPO-Ph resin/PETMP, the presence of unreactive impurities leads to the subsequent reduction of the enthalpy released. The formulation DGEBA/PETMP shows a similar degree of curing to the formulation with triepoxy phloroglucinol.

### 3.3. Characterization of Thermosets Prepared from 3EPO-Ph-Thiol Formulations 

The prepared thermosetting materials showed a good appearance and a high transparency. If we look at the *T*_g_s determined by calorimetry collected in Table 2, we can see that the increase in the functionality of the thiol has a beneficial effect on this parameter resulting into a temperature of 35 °C for TTMP and 55 °C for PETMP. Taking into account that compared to DGEBA, the use of the 3EPO-Ph resin has only a slight effect on the *T*_g_, both obtained materials present similar values as to the ones reported by us in a previous paper on DGEBA-thiol epoxy thermosets [29,30]. The partial substitution of TTMP by the aromatic thiol leads to an increase of 14 °C in the *T*_g_, although the functionality of TBBT is only two and therefore they only act as a chain extender.

**Figure 6:**
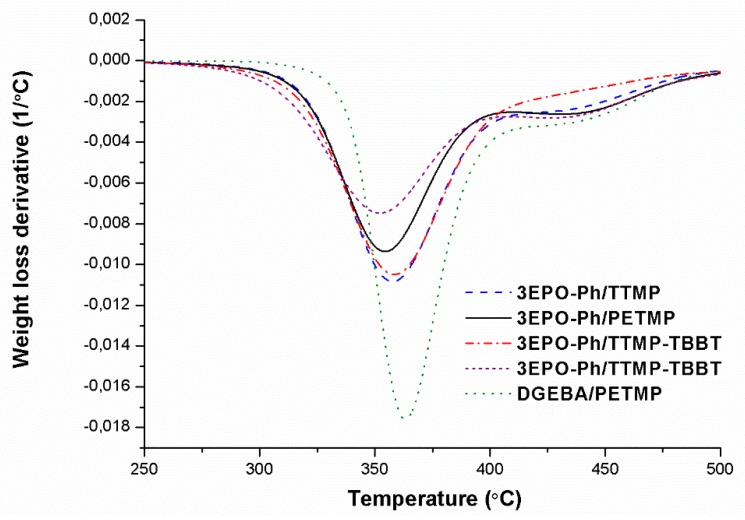
DTG curves under N_2_ at 10 °C/min of the materials obtained from the phloroglucinol derivative with different thiols.

The materials prepared were characterized by thermogravimetry to evaluate their stability at high temperatures. Figure 6 shows the derivative of the weight loss curves against temperature in inert atmosphere and Table 3 collects the main data obtained from the thermogravimetric studies. DGEBA derived materials were also tested for comparative purposes.

In Figure 6, it can be observed that all the studied thermosets follow a similar degradation pattern, with a main degradation process followed by weak degradation at high temperatures. This complex shape can be attributed to two different degradation mechanisms, the weight loss at lower temperatures is mainly due to the breakage of the thioether bonds, which are weak, and the second to the degradation of the ester bonds included in the thiol structure of PETMP and TTMP. In the material where TTMP has been partially replaced by the aromatic thiol without ester groups, the curve at higher temperature becomes smoother, as expected. The thermoset prepared from DGEBA begins to lose weight at higher temperature than those prepared from 3EPO-Ph, but once initiated the degradation, this proceeds faster. The lower rate of weight loss for phloroglucinol materials can be related to their higher crosslinked character, due to its trifunctionality. The higher crosslinking density provided by the 3EPO-Ph rings reduces the possibility of producing volatile fragments during the thermal decomposition process.

In Table 3, we can see that the initial degradation temperatures are quite similar for all the 3EPO-Ph thermosets, indicating that the same type of bond is initially broken. The higher temperature of initial degradation for DGEBA could be due to the high molecular weight of DGEBA aromatic moiety that delays the weight loss once the sulfur bonds are broken. The presence of the aromatic thioether moieties (from TBBT) does not affect the thermal stability of the materials, whereas the substitution of the pure triepoxy monomer by the corresponding resin slightly reduces its thermal stability.

**Figure 7:**
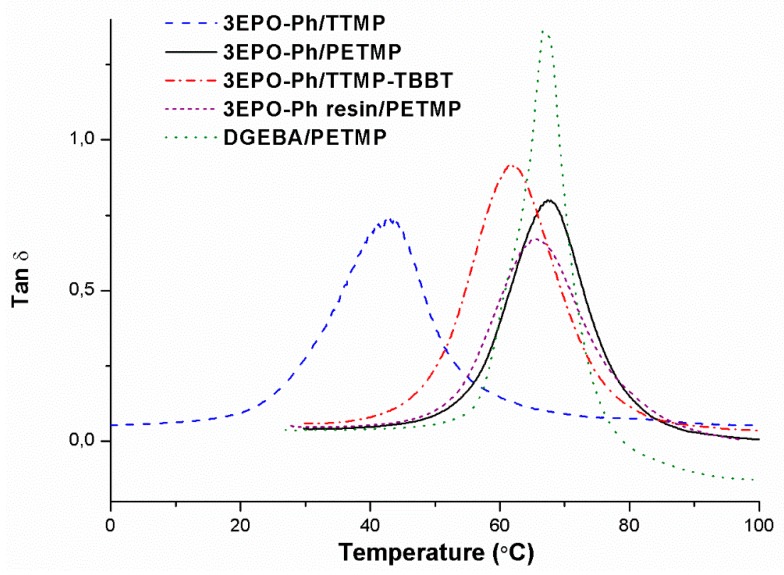
Plot of tan δ against temperature for the thermosets prepared.

The thermomechanical characteristics of the materials were tested by DMTA. Figure 7 shows the *tanδ* curves for the thermosets and the most relevant parameters extracted from these experiments are collected in Table 3.

If we look at the shape of these curves, we can see that they are monomodal, indicating that homogeneous networks have been formed, even in case of using a mixture of TTMP-TBBT thiols as curing agent. As we can see, the material from DGEBA shows a narrower and taller curve which is a sign of the best damping characteristics of all those materials. The temperatures of the maximum of the tan δ peak show the same trend than the *T*_g_s measured by DSC (see Table 2), but the values are higher because of the effect of the frequency on the α relaxation.

The moduli in the relaxed state also reflect the crosslinking density and the molecular weight between crosslinks. The relaxed moduli seem not to be affected by the purity of the epoxy compound, the materials obtained from 3EPO-Ph, and 3EPO-Ph resin are similar. The lowest value corresponds to the material crosslinked with TTMP-TBBT, with the highest molecular weight between crosslinks and quite similar to the thermoset prepared from DGEBA, which has the lowest crosslinking density.

If we look at the Young’s moduli in Table 3 we can see that the addition of TBBT to TTMP formulations leads to a notable increase in this characteristic, reaching the highest value achieved, due to the rigid structure of the aromatic moieties introduced. It should be mentioned that the low Young’s modulus for 3EPO-Ph/TTMP formulations is related to its low *T*_g_, near to the temperature at which this parameter has been determined. From the values in the table, we can also see that the use of the 3EPO-Ph resin increases the Young’s modulus in reference to the use of the purified monomer. The substitution of DGEBA by 3EPO-Ph slightly reduces the rigidity of these materials.

**Figure 8:**
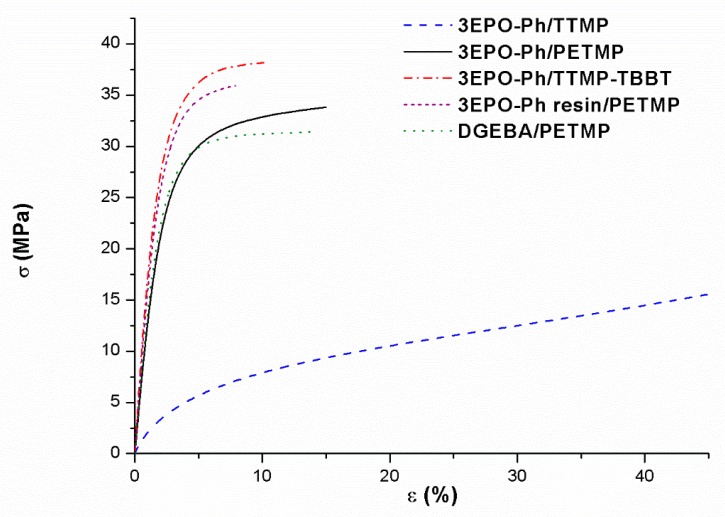
Stress-strain plot of tensile tests of the different materials prepared.

Dog-bone samples of cured materials were prepared to perform stress-strain tests in DMTA at 30 °C (see Figure 8). Table 4 shows the values obtained together with the values of microindentation hardness measured from the prismatic samples prepared for DMTA studies.

Figure 8 and mechanical data collected in Table 4 reveal that the materials prepared with PETMP show higher tensile stress, lower elongation at break and higher hardness than the thermoset prepared with TTMP, as expected from its higher crosslinking density. The tests have been performed at room temperature and therefore the 3EPO-Ph/TTMP material has already begun to relax. The addition of TBBT to TTMP cured materials is beneficial in these mechanical characteristics due to the rigidity introduced by its aromatic structure. The total substitution of the commercial resin DGEBA by renewable 3EPO-Ph monomer does not affect significantly the mechanical characteristics of these thermosetting materials. The values of the microindentation hardness are comparable in all materials with the only exception of the thermoset obtained from 3EPO-Ph/TTMP formulation, with a lower value, related to its low *T*_g_, near to the room temperature. The results obtained from stress-strain experiments and microindentation demonstrate slightly better performances for the renewable materials and the purification of 3EPO-Ph is not required to achieve good mechanical performance. According to the highest Young’s modulus measured in materials prepared from 3EPO-Ph resin, the use of the resin enhances tensile stress and reduces the elongation at break, but there is not a significant effect on hardness.

From the mechanical characterization of the renewable thermosets prepared, it can be stated that they are comparable and even slightly better than those obtained from the traditional DGEBA resin.

## 4. Conclusions

A simple synthetic route starting from phloroglucinol and reacting with a large excess of epichlorohydrin was used to obtain triglycidyl phloroglucinol (3EPO-Ph). The monomer was purified by column chromatography and its high purity confirmed by ^1^H- and ^13^C-NMR. It was used as renewable starting epoxy monomer in the preparation of new epoxy-thiol thermosets.

Triglycidyl phloroglucinol formulations with stoichiometric proportions of different thiols were prepared and subsequently cured in the presence of a basic catalyst. *N*,*N*-(4-dimethylamino pyridine), an encapsulated imidazole (LC-80) and 1-methylimidazolium tetrafluoroborate (BG) were tested, being BG the catalyst that shows the best latent characteristics. The curing, once initiated, is the fastest. The reactivity of these formulations highly depends on the thiol structure and on the purity of the epoxy compound and they are more reactive than the formulation prepared from DGEBA. The aromatic thiol (TBBT) reacts faster than aliphatic ones.

The thermosetting materials obtained show similar *T*_g_ values than those prepared with DGEBA and depend on the structure of the thiol employed. Little impurities in the 3EPO-Ph resin do not affect much the final characteristics of the thermosets.

The partial substitution of TTMP by an aromatic dithiol (TBBT) to 3EPO-Ph formulations increases significantly the mechanical performance of the thermoset and its *T*_g_ (from 35 to 49 °C).

Thermomechanical, stress-strain and microindentation characterizations of the thermosets prepared from phloroglucinol confirmed that this bio-based compound constitutes a good alternative to DGEBA.

## Figures and Tables

**Table 1:** Reaction enthalpies and *T*_g_s determined by DSC of the formulations 3EPO-Ph/PETMP with different catalysts studied.

Formulation	*Δh*^a^ (J/g)	*Δh*^b^ (kJ/eq)	*T*_g_^c^ (°C)
3EPO-Ph/PETMP/DMAP	578	128	56
3EPO-Ph/PETMP/LC-80	593	131	59
3EPO-Ph/PETMP/BG	583	130	55

^a^ Enthalpy released per gram of sample in a dynamic curing; ^b^ Enthalpy released per epoxy equivalent in a dynamic curing; ^c^ Glass transition temperature of the cured material.

**Table 2:** Calorimetric data from the curing of the different formulations, catalysed by 1 phr of BG.

Formulation	*Δh*^a^ (J/g)	*Δh*^b^ (kJ/eq)	*T*_g_^c^ (°C)
3EPO-Ph/TTMP	540	125	35
3EPO-Ph/PETMP	583	130	55
3EPO-Ph/TTMP-TBBT ^d^	521	113	49
3EPO-Ph resin/PETMP	536	118	53
DGEBA/PETMP	425	129	56

^a^ Enthalpy released per gram of sample in a dynamic curing; ^b^ Enthalpy released per epoxy equivalent in a dynamic curing; ^c^ Glass transition temperature of the cured material; ^d^ Formulation prepared with 60% of TTMP and 40% of TBBT in mol.

**Table 3:** Thermal and thermomechanical data of the thermosets prepared from 3EPO-Ph.

Sample	*T*_5%_^a^ (°C)	*T*_max_^b^ (°C)	*T*_tanδ_^c^ (°C)	*E*_r_^d^ (MPa)	*E*^e^ (MPa)
3EPO-Ph/TTMP	328	358	44	24	275
3EPO-Ph/PETMP	325	355	67	36	1992
3EPO-Ph/TTMP-TBBT^f^	328	359	62	20	2296
3EPO-Ph resin/PETMP	320	352	66	37	2584
DGEBA/PETMP	345	363	67	18	2158

^a^ Temperature of 5% of weight loss in N_2_ atmosphere; ^b^ Temperature of the maximum rate of degradation in N_2_ atmosphere; ^c^ Glass transition temperature determined by DMTA; ^d^ Storage modulus in the rubbery state determined at δ + 50 °C; ^e^ Young’s modulus at 30 °C under flexural conditions; ^f^ Material prepared from a formulation containing 60% of TTMP and 40% of TBBT in mol.

**Table 4:** Mechanical data from stress-strain and hardness testing of the thermosets prepared. Coefficients of variation less than 5% for stress, strain and tensile modulus.

Sample	Strain at Break ^a^ ε_b_ (%)	Stress at Break ^b^ σ_b_ (MPa)	Microindentation Hardness ^c,d^ (HV)
3EPO-Ph/TTMP	46.7	15.9	4.2 ± 0.3
3EPO-Ph/PETMP	15.0	33.8	10.1 ± 0.4
3EPO-Ph/TTMP-TBBT	7.9	35.9	9.8 ± 0.3
3EPO-Ph resin/PETMP	10.1	38.2	10.2 ± 0.3
DGEBA/PETMP	14.1	31.4	10.0 ± 0.8

^a^ Strain at break; ^b^ Stress at break; ^c^ Indentation test load of 10 g for formulations: 3EPO-Ph/TTMP; ^d^ Indentation test load of 25 g for formulations: 3EPO-Ph/PETMP; 3EPO-Ph/TTMP-TBBT and 3EPO-Ph resin/PETMP.

## References

[1] Mülhaupt R. (2013). Green polymer chemistry and bio-based plastics: Dreams and reality. Macromol. Chem. Phys..

[2] Kumar S., Krishnan S., Mohanty S., Nayak S.K. (2018). Synthesis and characterization of petroleum and biobased epoxy resins: A review. Polym. Int..

[3] Auvergne R., Caillol S., David G., Boutevin B. (2014). Biobased thermosetting epoxy: Present and future. Chem. Rev..

[4] Stemmelen M., Lapinte V., Habas J.P., Robin J.J. (2015). Plant oil-based epoxy resins from fatty diamines and epoxidized vegetable oil. Eur. Polym. J..

[5] Ding C., Shuttleworth P.S., Makin S., Clark J.H., Matharu A.S. (2015). New insights into the curing of epoxidized linseed oil with dicarboxylic acids. Green Chem..

[6] Fache M., Boutevin B., Caillol S. (2016). Vanillin production from lignin and its use as a renewable chemical. ACS Sustain. Chem. Eng..

[7] Savonnet E., Grau E., Grelier S., Defoort B., Cramail H. (2018). Divanillin-based epoxy precursors as DGEBA substitutes for biobased epoxy thermosets. ACS Sustain. Chem. Eng..

[8] Ma S., Liu X., Jiang Y., Tang Z., Zhang C., Zhu J. (2013). Bio-based epoxy resin from itaconic acid and its thermosets cured with anhydride and comonomers. Green Chem..

[9] Darroman E., Durand N., Boutevin B., Caillol S. (2016). Improved cardanol derived epoxy coatings. Prog. Org. Coat..

[10] Hu F., La Scala J.J., Sadler J.M., Palmese G.R. (2014). Synthesis and characterization of thermosetting furan-based epoxy systems. Macromolecules.

[11] Niedermann P., Szebényi G., Toldy A. (2015). Novel high glass temperature sugar-based epoxy resins: Characterization and comparison to mineral oil-based aliphatic and aromatic resins. Express Polym. Lett..

[12] Maisonneuve L., Lebarbé T., Grau E., Cramail H. (2013). Structure–properties relationship of fatty acid-based thermoplastics as synthetic polymer mimics. Polym. Chem..

[13] Vilela C., Rua R., Silvestre A.J., Gandini A. (2010). Polymers and copolymers from fatty acid-based monomers. Ind. Crops Prod..

[14] Nikafshar S., Zabihi O., Hamidi S., Moradi Y., Barzegar S., Ahmadie M., Naebe M. (2017). A renewable bio-based epoxy resin with improved mechanical performance that can compete with DGEBA. RSC Adv..

[15] Ramon E., Sguazzo C., Moreira P.M.G.P. (2018). A review of recent research on bio-based epoxy systems for engineering applications and potentialities in the aviation sector. Aerospace.

[16] Guzmán D., Ramis X., Fernández-Francos X., De la Flor S., Serra A. (2018). Preparation of new biobased coatings from a triglycidyl eugenol derivative through thiol-epoxy click reaction. Prog. Org. Coat..

[17] Guzmán D., Serra A., Ramis X., Fernández-Francos X., De la Flor S. (2019). Fully renewable thermosets based on bis-eugenol prepared by thiol-click chemistry. React. Funct. Polym..

[18] Chowdhury M.T.H., Bangoura I., Kang J.Y., Park N.G., Ahn D.H., Hong Y.K. (2011). Distribution of phlorotannins in the brown alga ecklonia cava and comparison of pretreatments for extraction. Fish. Aquat. Sci..

[19] Singh I.P., Bharate S.B. (2006). Phloroglucinol compounds of natural origin. Nat. Prod. Rep..

[20] Thomas N.V., Kim S.K. (2012). Handbook of Marine Macroalgae: Biotechnology and Applied Phycology.

[21] Yoon J.Y., Choi H., Jun H.S. (2017). The effect of phloroglucinol, a component of ecklonia cava extract, on hepatic glucose production. Mar. Drugs.

[22] Negrell C., Cornille A., de Andrade Nascimento P., Robin J.J., Caillol S. (2017). New bio-based epoxy materials and foams from microalgal oil. Eur. J. Lipid Sci. Technol..

[23] Noè C., Malburet S., Bouvet-Marchand A., Graillot A., Loubat C., Sangermano M. (2019). Cationic photopolymerization of bio-renewable epoxidized monomers. Prog. Org. Coat..

[24] Ménard R., Negrell-Guirao C., Ferry L., Sonnier R., David G. (2014). Synthesis of biobased phosphate flame retardants. Pure Appl. Chem..

[25] Ménard R., Negrell C., Fache M., Ferry L., Sonnier R., David G. (2015). From a bio-based phosphorus-containing epoxy monomer to fully bio-based flame-retardant thermoset. RSC Adv..

[26] Ng F., Bonnet L., David G., Caillol S. (2017). Novel biobased and food contact epoxy coatings for glass toughening applications. Prog. Org. Coat..

[27] Konuray O., Liendo F., Fernández-Francos X., Serra A., Sangermano M., Ramis X. (2017). Sequential curing of thiol-acetoacetate-acrylate thermosets by latent Michael addition reactions. Polymer.

[28] Konuray O., Areny N., Morancho J.M., Fernandez-Francos X., Serra A., Ramis X. (2018). Preparation and characterization of dual-curable off-stoichiometric amine-epoxy thermosets with latent reactivity. Polymer.

[29] Guzmán D., Ramis X., Fernández-Francos X., Serra A. (2014). New catalysts for diglycidyl ether of bisphenol A curing based on thiol–epoxy click reaction. Eur. Polym. J..

[30] Guzmán D., Ramis X., Fernández-Francos X., Serra A. (2015). Enhancement in the glass transition temperature in latent thiol-epoxy click cured thermosets. Polymers.

